# The effect of Tannic acid on colonic anastomosis in abdominal sepsis: An experimental study

**DOI:** 10.1371/journal.pone.0339175

**Published:** 2025-12-26

**Authors:** Oztekin Cikman, Sukru Tas, Omer Faruk Ozkan, Umut Ercan, Hakan Turkon, Nihal Kilic, Yilmaz Akgun, Muammer Karaayvaz

**Affiliations:** 1 Department of General Surgery, Faculty of Medicine, Yüzüncü Yıl University, Van, Turkey,; 2 Department of General Surgery, Çanakkale Onsekiz Mart University School of Medicine, Çanakkale, Turkey; 3 Department of General Surgery, Health Sciences University, Umraniye Research and Education Hospital, Istanbul, Turkey,; 4 Department of General Surgery, Ezine State Hospital, Çanakkale, Turkey,; 5 Department of Medical Biochemistry, Private Meddem Hospital, Isparta, Turkey; 6 Department of Medical Pathology, Faculty of Medicine, Çanakkale Onsekiz Mart University, Çanakkale, Turkey; Athens Medical Group, Psychiko Clinic, GREECE

## Abstract

**Background:**

This study aimed to evaluate the effect of tannic acid on colonic anastomosis in a sepsis model induced by cecal abrasion.

**Materials and methods:**

Thirty Sprague-Dawley rats were used. The animals were randomly divided into three groups of ten: Group 1 (n:10): Colonic anastomosis + 0.9% isotonic NaCl. Group 2 (n:10): Cecal ligation and puncture + Colonic anastomosis + 0.9% isotonic NaCl. Group 3 (n:10): Cecal ligation and puncture + Colonic anastomosis + Tannic Acid group. The rats were sacrificed on the fifth postoperative day, and the resected colon segments, bursting pressure, hydroxyproline levels, and histopathologic features of the anastomosis were evaluated.

**Results:**

The bursting pressure value was statistically significantly higher in Group 3, where tannic acid was administered (p < 0.05). Group 2 and Group 3, in which peritonitis was induced, had moderate levels of fibroblastic activity, inflammatory cell infiltration, neovascularisation, and collagen; whereas, they were higher in Group 1. Although the inflammation value dropped in Group 3 compared to Groups 1 and 2, there was no statistical difference in Group 2. The hydroxyproline values were 2.046 ± 1.1411 mcg/gr and 5.9730 ± 4.35900 mcg/gr tissues, respectively, in Groups 2 and 3, where septic conditions prevailed, and a statistically significant difference was found (p < 0.05).

**Conclusion:**

This experimental study revealed that the use of tannic acid during anastomosis has a positive effect on wound healing, acting through higher colonic anastomotic bursting pressures and higher tissue hydroxyproline levels.

## Introduction

Anastomotic leaks after colorectal surgery are still prognosed with high mortality and morbidity [1]. Postoperative complications associated with anastomotic leak lead to prolonged hospitalisation, elevated local recurrence rates, impaired quality of life, and a poor prognosis [2].

Anastomotic technique and meticulous work, colon bacteria, inflammation, blood supply, age, dietary condition, comorbidities, and medications are important factors for healing of colonic anastomosis and maintaining its integrity [3–5]. Intraperitoneal infections caused by colon bacteria delay wound healing by reducing the amount of tissue collagen and thus the mechanical strength of the anastomosis [6,7]. Therefore, primary anastomosis is avoided in case of intraperitoneal infection in both emergency and elective procedures, and multistage procedures are preferred [8].

Antibiotic prophylaxis, preoperative bowel preparation, and a faecal diversion with proximal ostomies are performed to prevent anastomotic leaks [9]. Furthermore, positive outcomes have been obtained in studies conducted with agents such as epidermal growth factor [10], carnitine [11], Ankaferd [12], and fibrin glue [13]. Today, various experimental and clinical studies have been ongoing for the early diagnosis and prevention of anastomotic leak after colorectal surgeries.

Tannins, a group of naturally formed polyphenol compounds, are commonly found in green tea and coffee, as well as fresh fruits such as pomegranate, dates and grapes, and many tree and plant species [14]. Tannic acid—the simplest hydrolysable tannin approved as a safe food additive by the US Food and Drug Administration (FDA) [14] and the European Union [15]—is one of the key components of conventional medicines [16].

Tannic acid has attracted great interest in recent years due to its extensive physiological effects, such as antioxidant, antitumor, antimicrobial, and anti-inflammatory effects, as well as its ability to interact with various proteins [17]. Tannic acid, which has been used in many studies, has neither been researched in anastomosis and abdominal sepsis nor has its role been discovered. The aim of this study is to assess how tannic acid affects colonic anastomosis in a sepsis model induced by cecal abrasion.

## Methods

### Ethical approval

This study was conducted at the Experimental Research Centre of Çanakkale Onsekiz Mart University with the approval of the Experimental Animals Ethics Committee of Çanakkale Onsekiz Mart University (no.2012/08–21) and the permission of the Directorate of the Experimental Research Centre of Çanakkale Onsekiz Mart University.

### Sample size, randomization, and blinding

The sample size was determined based on previous studies using similar colonic anastomosis models [18]. Animals were randomly assigned to experimental groups. Group allocation was performed by an investigator not involved in subsequent procedures to ensure allocation concealment. Burst pressure measurements and histopathological evaluations were conducted by investigators who were blinded to the treatment groups to minimize observer bias.

### Animals

A total of 30 Sprague-Dawley rats weighing between 200 and 250 g were used in the study. The rats were randomly divided into three groups of ten. Group 1 (n:10): Colonic anastomosis + 0.9% isotonic NaCl, Group 2 (n:10): Cecal ligation and puncture (CLP) + Colonic anastomosis + 0.9% isotonic NaCl, Group 3 (n:10): CLP + Colonic anastomosis + Tannic Acid.

During the study, the rules for the care and use of laboratory animals set out in the 1964 Declaration of Helsinki were strictly adhered to. The subjects were kept in standard cages with wood shavings on the bottom and a 12-hr light/12-hr dark cycle at a constant room temperature and humidity, one subject per cage throughout the study. During the experiment, the subjects were fed ad libitum with standard pellet rat feed and water.

### Experimental protocol

All rats in the experimental groups were anesthetized via intramuscular administration of 50 mg/kg Ketamine HCl (Ketalar® vial, 50 mg/mL, Eczacıbaşı, Istanbul, Turkey) and 10 mg/kg Xylazine HCl (Rompun® 2% vial, 20 mg/mL, Bayer AG, Leverkusen, Germany). Postoperative pain was monitored. Animals were observed twice daily for general condition, wound healing, and activity level. Humane endpoints were defined as severe distress, unresponsiveness, or >20% body weight loss.

Following anesthesia induction, the rats were placed in a supine position, and their abdominal skin was shaved. Antisepsis of the surgical site was ensured using povidone-iodine. A midline incision was made with tissue scissors to access the abdominal cavity by dissecting through the respective layers.

The cecum was isolated in Groups 2 and 3 and ligated with 3/0 silk suture at a distance corresponding to 20% of the total length of the colon, preserving intestinal passage. The antimesenteric side of the cecum was punctured three times with a 20-gauge needle. The cecum was squeezed slowly and gently to make sure that the punctures were clear. Cecal ligation, puncture and colon anastomosis was performed by a single surgeon.

The intestines and ligated cecum were carefully repositioned into the abdominal cavity. The abdominal wall was closed by separately suturing the fascia and skin using 4/0 silk sutures and sharp needles. For resuscitation, a subcutaneous injection of 0.9% NaCl solution (4 mL per 100 g of body weight) was administered. Group 1 underwent laparotomy without cecal ligation or puncture. Postoperative administration of antibiotics and analgesics was not performed for any of the rats in the study.

Twenty-four hours after the initial surgical procedure, all rats received a single systemic dose of 50 mg/kg Sodium Ampicillin + Sulbactam (Combicid, Bilim İlaç, Istanbul, Turkey), followed by relaparotomy under general anesthesia. This approach was based on the model described by Wang et al. [18], aiming to mimic the therapeutic antibiotic administration applied by surgeons upon detection of abdominal sepsis.

To confirm the presence of intra-abdominal sepsis, a peritoneal fluid sample was aseptically collected from the rats using a sterile swab. Following this, peritoneal lavage was performed with 40 mL of warm 0.9% NaCl solution. Subsequently, a full-thickness incision was created in the colonic segment, located 3 cm proximal to the peritoneal reflection of the left colon.

The distal and proximal ends were then anastomosed end-to-end in a single layer with Gambee sutures using a round needle 4/0 prolene. The intestines were repositioned into the abdominal cavity. The abdomen was closed by suturing the fascia and skin separately with 4/0 silk and sharp needles.

The rats in Groups 1 and 2 were injected with 0.9% isotonic NaCl at a dose of 15 mL/kg intraperitoneally, and the rats in Group 3 were injected with tannic acid (Tannen, ITK, Izmir, Turkiye) at a dose of 10 mg/kg intraperitoneally. 24 hours after the first dose, Group 1 rats were injected with 0.9% isotonic NaCl at a dose of 15 mL/kg intraperitoneally and Group 3 rats were injected with tannic acid at a dose of 10 mg/kg intraperitoneally for 5 post-operative days. On the fifth postoperative day, rats in all groups were sacrificed under high-dose anaesthesia (100 mg/kg thiopental sodium), and relaparotomy was performed. The degrees of intra-abdominal adhesion were examined. The colonic segment that was anastomosed in the previous surgery was resected by including 2 cm of healthy colonic tissue proximal and distal to the anastomosis line.

The measurement test for anastomotic bursting pressure was first run in the colonic segment obtained from the groups. Then, the colonic segment was divided into two parts to enclose the anastomosis line in both parts. The initial portion was placed in an Eppendorf tube containing a 10% formaldehyde solution and stored at +4°C. The second portion was transferred to an Eppendorf tube containing 0.9% NaCl solution and stored at −80°C.

### Microbiology

An intraabdominal fluid sample was collected all animals using a sterile swab to prove the presence of intraabdominal sepsis. The sample was inoculated in 5 ml of BHIB (Brain Heart Infusion Broth) and incubated at 37°C for 24 hours. Afterwards, the sample was passaged onto 5% sheep blood agar and EMB agar and incubated once again at 37°C for 24 hours. The microorganisms grown were identified by conventional microbiological methods.

While bacterial growth was detected in the intraabdominal fluid cultures of all animals in Groups 2 and 3, all cultures obtained from the control group (Group 1) were negative. Among the cultures showing growth, Gram-negative bacilli were isolated in approximately 90% of the samples, and mixed bacterial growth was frequently observed in the experimental groups.

### Measurement of anastomotic bursting pressure

The proximal and distal ends of all resected intestinal segments were tightly ligated with 3/0 silk by inserting a serum set into the lumen. The serum set at the proximal end was attached to the infusion pump, and the serum set at the distal end was connected to the monitor for pressure measurement, and the necessary mechanism was set up to display the intraluminal pressure in millimetres of mercury (mmHg).

The anastomosis line was inserted into a container filled with water, and air was supplied into the lumen continuously at a flow rate of 4 ml/min. The first air outflow from the anastomosis line was recorded as anastomotic bursting pressure.

### Histopathological analysis

All histopathological evaluations were performed at the Department of Pathology, Çanakkale Onsekiz Mart University Research and Application Hospital. Tissue samples, fixed in 10% formalin, underwent standard histological processing. Paraffin-embedded tissue blocks were sectioned at 3 μm thickness and stained with Hematoxylin and Eosin (H&E) for microscopic examination.

A single pathologist, blinded to the study groups, performed all evaluations using a light microscope (Carl Zeiss Axioscope) and captured digital images with a high-resolution imaging system (Carl Zeiss Axiocam ICc3 3.3 MP digital camera, Carl Zeiss Axiovision software). Representative images for each group were included in the revised figures with scale bars.

The anastomotic line was evaluated using the Ehrlich–Hunt scoring system [19], which considers four histological domains: Inflammatory cells, Fibroblasts, Neovascularization, Collagen deposition.

Each domain was scored on a scale from 1 to 4 (Table 1). The composite score was calculated as the sum of the four domain scores.

**Table 1:** Model of Ehrlich-Hunt.

Stage	Inflammatory cells/fibroblasts/neovascularization/collagen
1	Small amount but present in a scattered pattern
2	Small amount and present everywhere
3	High amount but present in a scattered pattern
4	High amount and present everywhere

### Determination of tissue hydroxyproline level

Hydroxyproline levels in the tissue samples were quantitatively analyzed using a spectrophotometric approach with a commercial BioVision Hydroxyproline Assay Kit (Cat. No. K555-100, Milpitas, CA, USA). Intestinal tissue specimens were preserved at −80°C in an ultra-low temperature freezer until analysis. Tissue samples containing the anastomotic line were homogenized in the laboratory utilizing a homogenizer (Wiggenhauser, D-130, Germany) at a ratio of 100 μL of distilled water per 10 mg of tissue. Subsequently, 100 μL of 12N HCl was added to an equal volume (100 μL) of the homogenate, followed by hydrolysis at 120°C for 3 hours.

Following hydrolysis, 10 μL of the processed sample was transferred to a 96-well flat-bottom microplate and allowed to dry completely. Subsequently, 100 μL of an oxidation buffer containing chloramine T was added to each well, and the plate was incubated at room temperature for 5 minutes. Thereafter, 100 μL of dimethylaminobenzaldehyde (DMAB) was introduced into each well, followed by incubation at 60°C for 90 minutes in a controlled heating environment. Upon completion of the incubation period, the samples were cooled to room temperature, and absorbance measurements were recorded at 560 nm using an automated microplate reader (Tecan Infinite 200 PRO, Tecan Group Ltd, Männedorf, Switzerland). The hydroxyproline concentration was subsequently quantified and expressed as micrograms per gram (μg/g) of tissue.

### Statistical analysis

All data collected through subject follow-up forms were analyzed using IBM SPSS Statistics 20. The normality of the data was assessed both visually (histograms and Q–Q plots) and analytically (Shapiro–Wilk test), considering the sample size.

Variables that met the assumption of normal distribution were analyzed using one-way ANOVA, followed by Tukey’s post-hoc test for pairwise comparisons when homogeneity of variance was confirmed (Levene’s test, p > 0.05). When variances were not homogeneous, Welch ANOVA was applied, followed by Tamhane’s T2 post-hoc test.

For variables that did not meet the normality assumption, comparisons among groups were performed using the Kruskal–Wallis test, and pairwise comparisons were conducted using Dunn’s test with Bonferroni correction.

Descriptive statistics are presented as mean ± standard deviation (SD) for normally distributed variables and as median (interquartile range) for non-normally distributed variables. The significance level was set at p < 0.05.

Effect sizes (η² for parametric tests and ε² for non-parametric tests) and corresponding 95% confidence intervals are reported for all main outcomes.

## Results

None of the animals that were included in the study died during the surgery or during the collection of the samples.

### Bursting pressure

The anastomoses of the subjects in all groups burst during the measurement of bursting pressure, and the obtained burst pressure values were statistically analysed by the One-Way ANOVA test. When the values in the groups were examined, the mean values were found to be 233 ± 9.695 mmHg in Group 1, 200.30 ± 9.967 mmHg in Group 2, and 219.90 ± 16.272 mmHg in Group 3 (Table 2). The bursting pressure value was statistically significantly higher in Group 3, where tannic acid was administered (p < 0.05).

**Table 2:** Pairwise comparison of groups and p-values according to bursting pressure, histopathological score, and hydroxyproline values.

Groups	Bursting Pressure (mmHg)	Histopathological Score	Hydroxyproline Level (mcg/gr tissue)
Group I	233 ± 9.695	13.30 ± 0.823	5.593 ± 3.6009
Group II	200,30 ± 9.967 a	9.50 ± 0.707 a	2.046 ± 1.1411 b
Group III	219.90 ± 16.272 c,e	6.80 ± 3.584 d,e	5.9730 ± 4.35900 d,e

a, c p < 0,05 compared to group 1. ^b,d^ P > 0,05; compared to group 1. ^e^ p < 0,05 compared to group 2.

Data represent means+SD.

### Histopathological analysis

When the histopathological staging results of the subjects were analysed according to the Ehrlich-Hunt model, the mean values were found to be 13.30 ± 0.823 in Group 1, 9.50 ± 0.707 in Group 2, and 6.80 ± 3.584 in Group 3 (Table 2).

According to the histopathological staging conducted based on the Ehrlich-Hunt model to evaluate wound healing at the anastomosis line, all parameters (fibroblastic activity, inflammatory cell infiltration, neovascularisation, and collagen) measured in Groups 2 and 3, where peritonitis was induced by cecal ligation and puncture, appeared to be moderate; whereas they were high in Group 1, where neither cecal ligation nor puncture was performed (Table 2). According to histopathological analysis, the inflammation value dropped in Group 3 compared to Groups 1 and 2; whereas, there was no statistically significant difference in Group 2. Representative microscopic images used for histopathological evaluation are provided in S1 Fig - S3 Fig.

### Hydroxyproline level

When hydroxyproline levels of the subjects were analysed, the mean values were found to be 5.593 ± 3.6009 in Group 1, 2.046 ± 1.1411 in Group 2, and 5.9730 ± 4.35900 in Group 3 (Table 2). The hydroxyproline values were 2.046 ± 1.1411 mcg/gr and 5.9730 ± 4.35900 mcg/gr tissues, respectively, in Groups 2 and 3, where septic conditions prevailed, and a statistically significant difference was found (p < 0.05).

## Discussion

In colorectal surgery, achieving uncomplicated healing of anastomoses remains a significant clinical challenge. [20]. Primary colonic anastomoses are less preferred by surgeons, and multistep procedures are followed, especially when factors such as peritonitis that threaten anastomotic safety are present [21]. Currently, the effects of pharmacological agents are being investigated to enable safe colonic anastomosis in infected abdomens during colorectal surgery. The present study revealed that tannic acid can be used to make primary anastomosis safer in the presence of peritonitis, which adversely affects anastomotic healing.

Tannic acid has been shown to effectively attenuate intestinal damage caused by oxidative stress and inflammatory responses and to reinforce intestinal barrier function [16]. Furthermore, its inhibitory effects on various microorganisms, especially human intestinal bacteria, have been shown [22]. Snoussi et al., reported that tannic acid inhibited the growth of Gr (-) bacteria [23]. On the other hand, Romero-García et al., showed that tannic acid inhibited E. coli by eliminating their ability to form biofilm [24]. In their study, Taleb et al., [25] reported that polyphenols delayed bacterial growth by producing hydrogen peroxide, which mediates bacterial growth inhibition caused by oxidative stress, thereby exhibiting antibacterial activity. The present study also showed that administration of tannic acid intraperitoneally, both in the presence of peritonitis and in anastomosis performed in the uninfected abdomen, positively affected healing and improved the safety of anastomosis.

When anastomosis is performed in the gastrointestinal system, inflammation develops in response to traumatic injury and foreign materials such as sutures. The inflammatory cells in the medium promote collagenosis in the perianastomotic area through proteolytic enzymes. Therefore, the anastomotic healing is delayed in the presence of intra-abdominal infection [3]. Although the present study indicated that fibroblastic activity, inflammatory cell infiltration, neovascularisation, and collagen were at moderate levels in Groups 2 and 3, in which peritonitis was induced, none of these parameters were found at high levels in Group 1. According to histopathological analysis, although the inflammation value dropped in Group 3 compared to Groups 1 and 2, there was no statistically significant difference in Group 2. This outcome is thought to be associated with the anti-inflammatory effects of tannic acid. Previous studies have demonstrated that tannic acid reduces the inflammatory response by inhibiting the production of pro-inflammatory mediators, particularly in immune cells. Based on these properties, tannic acid is considered to contribute positively to the wound healing process.

The parameter that best shows anastomotic healing early on is anastomotic bursting pressure. Anastomotic bursting pressure is a mechanical evaluation method which shows the resistance of colonic anastomosis to the increase in intraluminal pressure [26]. Anastomotic bursting pressure tests have shown that the force to be applied gradually escalates from the third day of anastomosis and peaks in one week [21]. Since the bursting pressure was considered to have reached a sufficient level, the rats were sacrificed on the fifth post-operative day, and the measurement test for anastomotic bursting pressure was run in the colon segment obtained. In this study, the bursting pressure value was statistically significantly higher in Group 3, where tannic acid was administered (p < 0.05).

Anastomotic healing is the recovery of tissue strength to pre-anastomotic levels, and this depends on the quality and amount of newly synthesised collagen [27]. Collagen, which plays an important role in all phases of wound healing, contains large amounts of hydroxyproline. High hydroxyproline levels, higher fibroblast activation, and collagen fibre ratios are shown as histopathological evidence for safe anastomosis [3,28]. In the present study the hydroxyproline content was determined in the anastomotic tissue by means of collagen synthesis and wound healing parameters and it was observed that hydroxyproline levels significantly elevated in Groups 2 and 3, where septic conditions prevailed, compared to Group 1.

A limitation of this study is the lack of mechanistic markers such as MPO, IL-6, and MMP-9, which could have provided insight into inflammatory and tissue remodeling processes. Additionally, the degree of peritonitis during the anastomotic procedure was not assessed, which may have influenced the healing outcomes. These limitations should be considered when interpreting the findings.

## Conclusion

In conclusion, this experimental study demonstrated that intraperitoneal administration of tannic acid provided a positive effect on colonic anastomotic wound healing, as suggested by increased bursting pressure and higher tissue hydroxyproline levels. However, the clinical implications of these experimental findings remain unknown and require further investigation in future studies.

## Supporting information

S1 Fig Intense fibroblastic activity, inflammatory infiltration, and vascular proliferation (H&E × 40). (PDF)

S2 Fig Intense fibroblastic activity, inflammatory infiltration, and vascular proliferation at higher magnification (H&E × 100). (PDF)

S3 Fig Minimal fibroblastic activity, inflammatory infiltration, and limited vascular proliferation under the mucosa (H&E × 40). (PDF)
