# Supplementary material for: The effect of Tannic acid on colonic anastomosis in abdominal sepsis: An experimental study
Source: PLoS One. 2025 Dec 26;20(12):e0339175. doi: 10.1371/journal.pone.0339175 (PMC12742727; doi:10.1371/journal.pone.0339175)

Figure 2: Intense fibroblastic activity, inflammatory cell infiltration, and vascular proliferation of different sizes are shown at high magnification (H&Ex100).

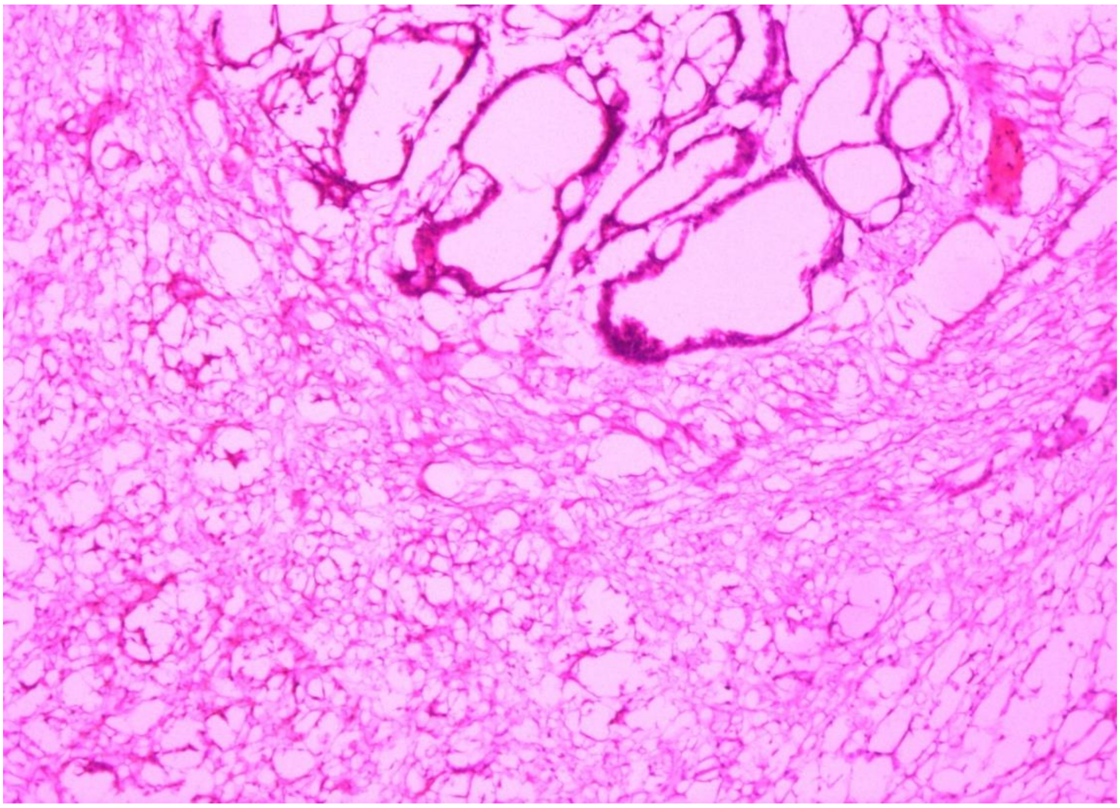

Supplement: S2 Fig — (PDF) [file pone.0339175.s002.pdf]
