# Supplementary material for: The effect of Tannic acid on colonic anastomosis in abdominal sepsis: An experimental study
Source: PLoS One. 2025 Dec 26;20(12):e0339175. doi: 10.1371/journal.pone.0339175 (PMC12742727; doi:10.1371/journal.pone.0339175)

Figure 3: Minimal fibroblastic activity, inflammatory cell infiltration, and a few vascular proliferations under the mucosa are visible under microscopic observation (H&Ex40).

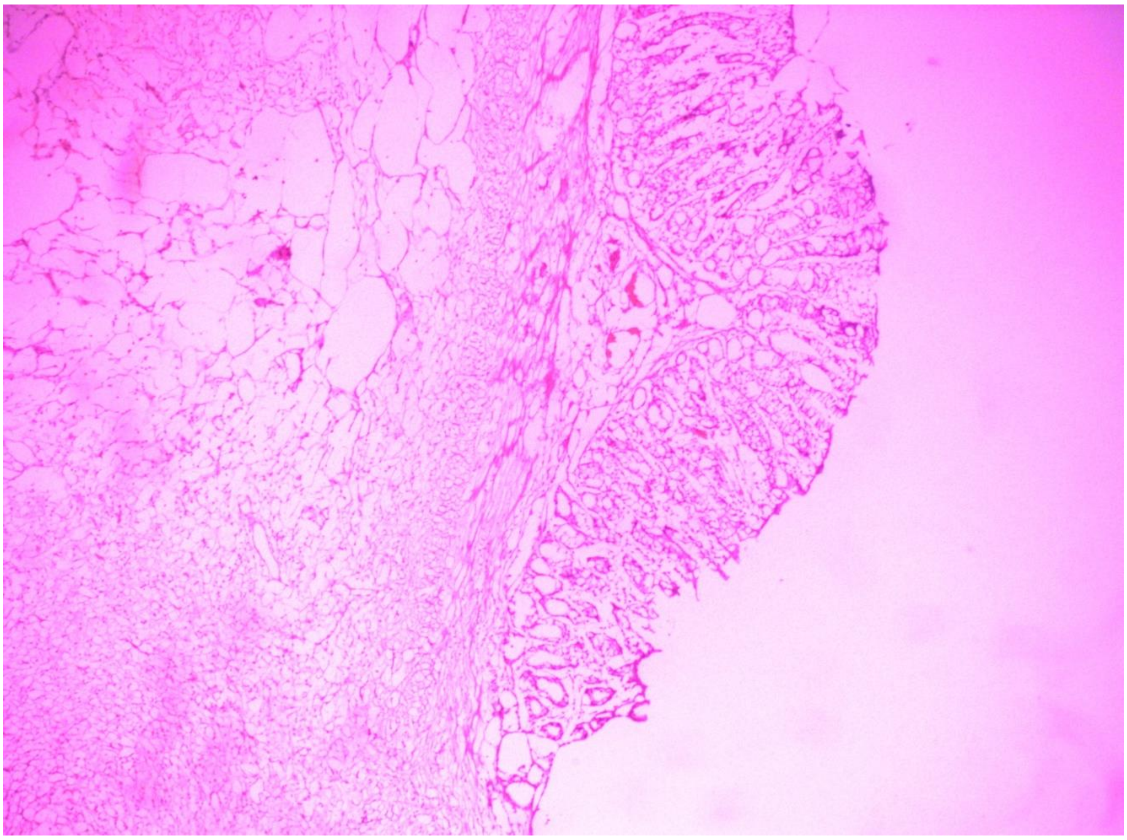

Supplement: S3 Fig — (PDF) [file pone.0339175.s003.pdf]
